# A Bibliometric Analysis of Research Progress on Age-Related Macular Degeneration and Autophagy From 2010 to 2024

**DOI:** 10.1155/joph/6670966

**Published:** 2025-12-02

**Authors:** Nana Meng, Leizhou Xia, Yiqing Gong, Chunhe Shi, Peirong Lu

**Affiliations:** ^1^Department of Ophthalmology, The First Affiliated Hospital of Soochow University, Suzhou, Jiangsu, China; ^2^Department of Ophthalmology, Affiliated People's Hospital, Jiangsu University, Zhenjiang, Jiangsu, China; ^3^Department of Ophthalmology, Zhenjiang Kangfu Eye Hospital, Zhenjiang, Jiangsu, China; ^4^Department of General Surgery, Affiliated People's Hospital, Jiangsu University, Zhenjiang, Jiangsu, China

**Keywords:** age-related macular degeneration (AMD), autophagy, bibliometric analysis, mitophagy, oxidative stress

## Abstract

**Background:**

Autophagy regulates intracellular metabolism and is crucial in the development of age-related macular degeneration (AMD). Despite the growing number of studies on AMD and autophagy in recent years, bibliometric analyses in this field remain scarce. Therefore, a bibliometric analysis was applied to explore the research trends and hot spots of this field in this study.

**Methods:**

We collected publications on autophagy in AMD from the MEDLINE database, covering the period from January 2010 to October 2024. The “bibliometrix” R package (Version R 4.2.3) was utilized for bibliometric analysis, and WPS Excel, PowerPoint, and Word (12.1.0.18276) were used to manage data and create related tables.

**Results:**

A total of 349 articles were included. The amount of literature was on the rise from 2010 to 2024. China leads in article quantity, whereas the United States holds the most influence. Although Finland ranks the third position in publication volume, followed by China and the United States, Finland led research in this field, with the University of Eastern Finland being the most active and prolific institution and Kaarniranta Kai as the most productive and influential author. International Journal of Molecular Sciences and Autophagy is the journal with the most volume. The three most referenced studies primarily examine the interplay between inflammation, oxidative stress, and autophagy in retinal pigment epithelial cells. The analysis for keywords found that mitophagy has also received increasing attention in this field.

**Conclusions:**

This bibliometric analysis identifies current research hotspots in autophagy related to AMD and informs future research directions. Future trends in this field may involve identifying and developing novel autophagy-targeted therapies for the prevention and treatment of AMD.

## 1. Background

Age-related macular degeneration (AMD) is a retinal disease characterized by progressive central visual function impairment and is the leading cause of severe vision loss and blindness in people over 50 in developed countries [1, 2]. By 2040, the global population of AMD patients is expected to reach around 288 million [3]. Currently, there are over 6 million AMD patients in China. The prevalence of AMD is anticipated to increase due to economic development and higher life expectancy [2]. AMD is clinically categorized into two types: dry (non-neovascular or atrophic) AMD and wet (neovascular) AMD (nAMD) [4]. Initial clinical indicators in both forms involve macular pigment irregularities and the buildup of lysosomal lipofuscin and extracellular drusen deposits, which progressively advance to geographic atrophy (GA) or choroidal neovascularization (CNV). Dry AMD accounts for about 80%–90% of all AMD patients, yet there are currently no effective treatment options [5]. Additionally, dry AMD can convert to nAMD, the risk of developing nAMD in eyes with GA is 29% after 4 years [6]. Although wet AMD affects just 10%–15% of individuals with AMD, it causes 90% of the severe vision loss associated with the condition [5]. Antivascular endothelial growth factor (anti-VEGF) is currently the main treatment. Although most patients can achieve improved vision and quality of life, about one-third patients still have no obvious effect after anti-VEGF treatment [7–9]. Furthermore, the requirement for lifelong repetitive injections elevates both the economic burden on patients and society and the risk of intraocular infection [10, 11]. Therefore, there is an urgent need to explore economical, safe, and effective treatment measures to prevent and/or treat AMD.

At present, the specific pathogenesis of AMD has not been fully defined. More and more studies have shown that AMD is a degenerative fundus lesion involving many factors such as age, race, smoking, drinking, heredity, metabolic disorders, oxidative stress, and immune inflammation [12]. Impaired lysosomal clearance and the accumulation of waste material in retinal pigment epithelial (RPE) cells are key cellular contributors to AMD [13]. Autophagy is crucial for cellular waste management and significantly influences AMD development [14]. Autophagic flux levels are reduced in RPE cells from AMD donors compared to those from healthy controls [15]. Mitter and colleagues exposed adult RPE (ARPE) Cell Line-19 cells to H_2_O_2_ and measured changes in autophagic activity, showing that autophagy was stimulated in the acute phase but reduced by chronic oxidative stress. A similar phenomenon was confirmed in the Sod2 knockdown mouse model: Autophagic activity increases in the early stage but is impaired at later stages of AMD in this model [13]. Modulating autophagy pathways has emerged as a potential strategy approach for AMD. Rapamycin-induced autophagy upregulation reduced oxidative stress and reactive oxygen species generation, while 3-methyladenine–mediated autophagy inhibition increased cell susceptibility to H_2_O_2_-induced toxicity and death [13]. Moreover, upregulated autophagy can inhibit the production of inflammatory factors, while downregulated autophagy could increase inflammatory injury and even cause apoptosis [16]. A thorough grasp of the interaction between autophagy and AMD is essential for uncovering their mechanisms and developing potential treatments.

Recently, numerous studies have increasingly concentrated on autophagy in AMD. Traditional analytical methods are insufficient for thoroughly evaluating the development of this research field due to the surge in publications. Bibliometrics employs mathematical and statistical techniques to quantitatively analyze the academic literature within a specific research field. It aids scholars in understanding the current state of development, identifying research hotspots, and forecasting future research trends in this field [17]. Bibliometric analysis has been previously employed to assess the hot spots and key areas in retinal diseases and autophagy [18]. However, there is still a lack of in-depth bibliometric analysis of autophagy in AMD. This study is the first to employ bibliometric methods to evaluate the status and trends of autophagy in AMD, aiming to guide future research.

## 2. Methods

### 2.1. Data Source and Retrieval Strategy

**Figure 1:**
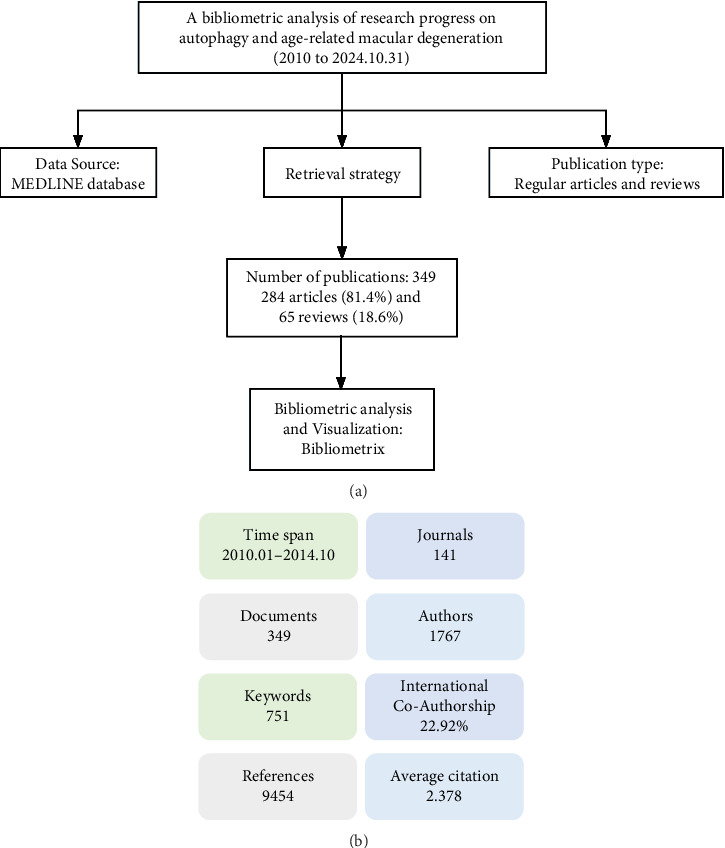
Diagram outlining the literature retrieval and analysis process, alongside statistical evaluation of data concerning age-related macular degeneration (AMD), and autophagy. (a) Flowchart of the retrieval and analysis process on AMD and autophagy from 2010 to 2024 in the MEDLINE database. (b) Statistical analysis of overall data including timespan, journals, documents, authors, keywords, international co-authorship, references, and average citation per document.

The bibliometric analysis in this study utilized the MEDLINE database, which provides abstracts and citations from over 4300 biomedical journals across more than 70 countries since 1966. It covers almost all areas of medicine and is the biomedical database used most frequently by medical professionals [19]. The MEDLINE database was searched for the period from January 1, 2010, to October 31, 2024. Several prechecks were conducted to refine the search formula, enhancing the accuracy and comprehensiveness of the final search results. The final retrieval strategy employed was (macular degeneration OR age-related macular degeneration OR AMD OR retinal macular degeneration OR macular degeneration disease) AND (autophagy OR autophagic process OR autophagic pathway OR autophagic degradation). The publication is in English and categorized as either an article or a review.  Figure 1(a) illustrates the detailed flowchart for the literature retrieval strategy employed in this study. A total of 349 publications were retrieved from the MEDLINE database for this study.

### 2.2. Bibliometric Analysis

This study utilized the “bibliometrix” R package (Version R 4.2.3) for bibliometric analysis, creating a global literature distribution map and extracting data on key journals, authors, citations, keywords, institutions, countries, and co-occurrence networks. The “PubMed export file” format was used to import full metadata from the MEDLINE database. Initial data interpretation involved the “biblioAnalysis ()” and “summary ()” functions to assess the trends in annual article distribution, document types, publication year averages, citation rates, co-authorship metrics, document authorship, prolific authors, highly cited manuscripts, corresponding author countries, publication and citation counts by country, and influential sources and keywords. Network analysis was conducted using “metaTagExtraction” and “Biblionetwork,” visualized with “Networkplot.” The “Biblioshiny ()” tool was employed for further analyses, including national and institutional collaboration networks, keyword analysis, co-occurrence network integration, and topic mapping. In addition, WPS Excel, PowerPoint, and Word (12.1.0.18276) were used to manage data and create related tables.

## 3. Results

### 3.1. Analysis of Articles Stratified by Publication Year

According to the retrieval strategy and analysis process, a total of 349 publications were obtained from January 2010 to October 2024 that met the study's criteria, comprising 284 articles (81.4%) and 65 reviews (18.6%) (Figure 1(a)). Besides, a brief description of the statistical analysis of all the included publications is shown in Figure 1(b). The 349 publications come from 141 academic journals, involving 1767 researchers, 751 keywords, and 9454 references, with the international co-authorship 22.92% and the average citation 2.378 (Figure 1(b)).

**Figure 2:**
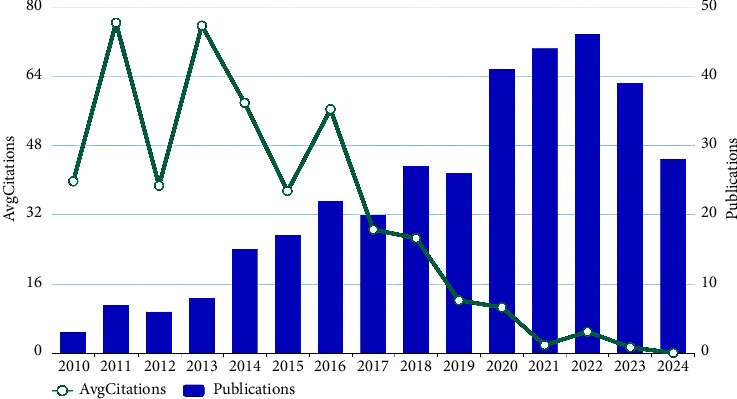
The annual publication count and mean citation rate for research on autophagy in AMD from 2010 to 2024.

Figure 2 demonstrates an annual increase in publications concerning AMD and autophagy. Between 2010 and 2013, publication output was minimal, indicating stagnation in research activity. The number of publications increased steadily from 2014 up to 2019, which indicated that this topic has begun to receive attention. Between 2020 and 2024, publications on autophagy in AMD surged, peaking in 2022, revealing that the research has reached a mature stage.

Figure 2 also depicts the trend in the annual average citations. In 2011, the number of annual average citation reached its highest point at 75.82. Between 2010 and 2018, citation counts exhibited moderate variability, with values ranging from 25.73 to 75.82. However, there has been a precipitous decrease in the average citation counts since 2019, which has plummeted to 1.53 by 2024. This phenomenon is a well-known manifestation of the “citation lag effect” in bibliometrics, whereby recent publications have not yet had sufficient time to accumulate citations. Therefore, this declining trend should be regarded as a natural consequence of the data collection point, rather than direct evidence of higher quality of earlier publications (2010–2018). To objectively compare the impact of publications from different periods, standardized metrics with a fixed time window or a longer observation cycle are required.

### 3.2. Analysis of Countries/Regions

**Figure 3:**
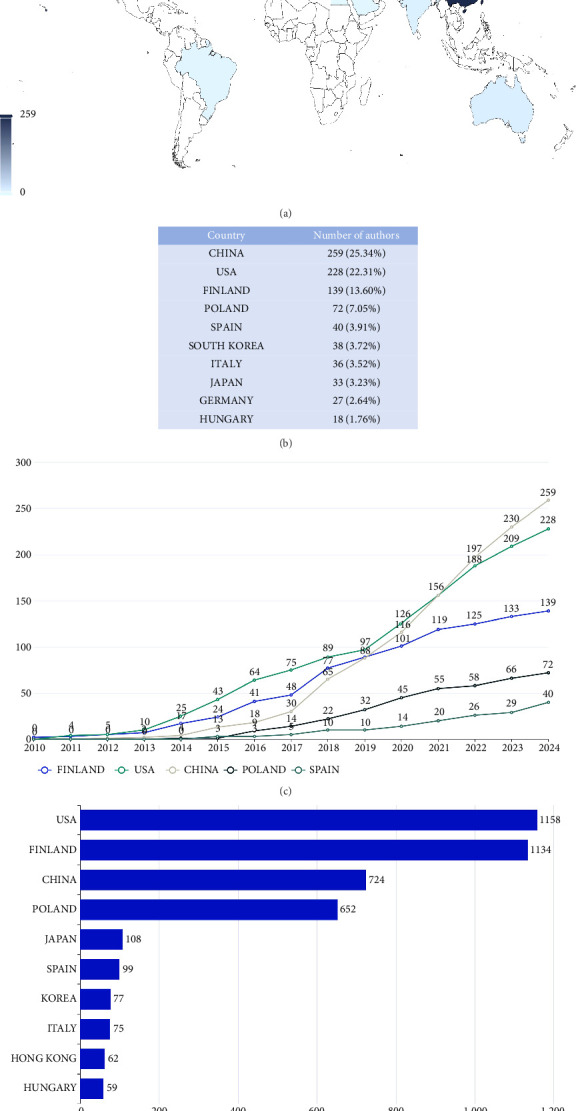
Analysis of countries/regions on AMD and autophagy. (a) The global distribution of contributing authors on AMD and autophagy from 2010 to 2024. (b) The top 10 countries/regions ranked by the number of authors. (c) The annual trend in contributing authors on autophagy and AMD (2010–2024) for the top five countries/regions. (d) The top 10 countries/regions according to the national citations.

The 349 publications were contributed to by 1767 total authors. After removing duplicates, the analysis identified 1022 unique authors from 33 countries/regions. The global distribution of this author base is mapped in Figure 3(a), and Figure 3(b) displays the top 10 countries in terms of their respective number of authors. The authors from China formed the largest contingent (259 authors, 25.34%), demonstrating a dominant research capacity in this field, followed closely by authors from the United States (228 authors, 22.31%) and Finland (139 authors, 13.60%). Collectively, the authors from China and the United States accounted for approximately 50% of the total research contribution, indicating a substantial collective focus on this field.

Figure 3(c) illustrates the annual trend in contributing authors on autophagy in AMD from 2010 to 2024 for the top five countries/regions. The United States consistently emerged as the foremost contributor, leading in the number of authors from 2010 to 2021, after which China assumed the leading role. This dynamic shift reflects changes in global research engagement over time. However, the global ranking of national citations does not align with the national output rankings. As shown in Figure 3(d), the United States ranks the first with a cumulative total of 1158 citations. Finland ranks the second place with a cumulative total of 1134 citations, yet China only ranks third with 724 citations.

### 3.3. Analysis of Institutions

**Figure 4:**
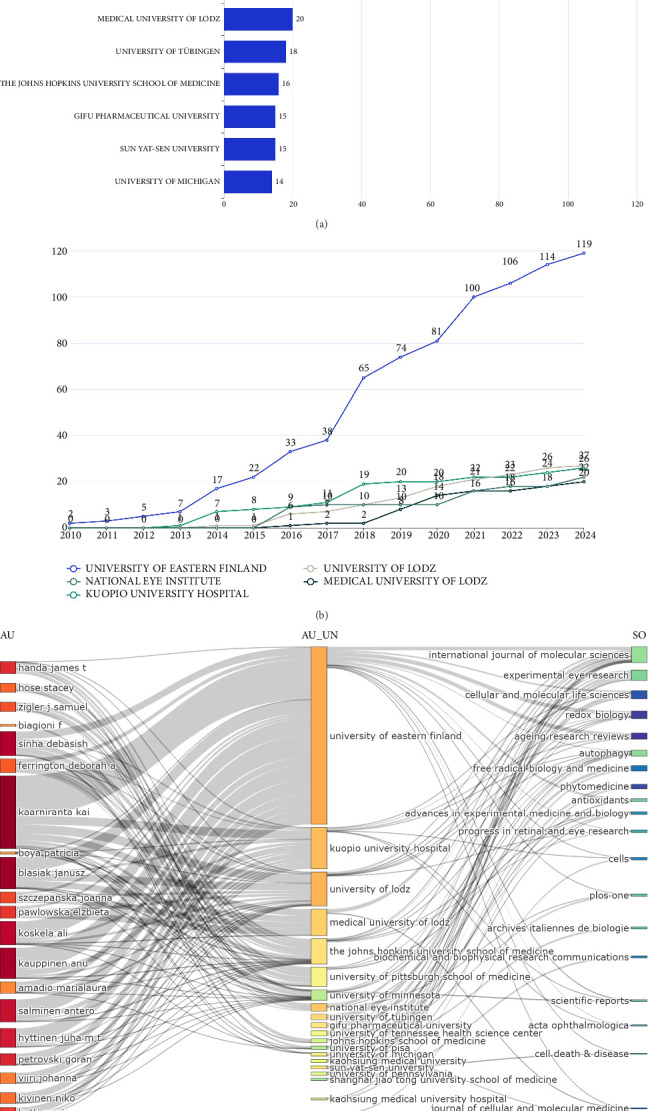
Analysis of institutions on AMD and autophagy. (a) The leading 10 institutions in research productivity on AMD and autophagy from 2010 to 2024. (b) The research output over time for the leading five institutions in the study of AMD and autophagy. (c) A three-field plot analyzing authors (AU), institutions (AU_UN), and academic journals (SO) related to AMD and autophagy.

Figure 4(a) lists the top 10 institutions with the highest research productivity on AMD and autophagy from 2010 to 2024. The University of Eastern Finland published the most research papers (*n* = 119), which is an overwhelming lead over the second-placed University of Lodz (*n* = 27), 4.4 times as many as Lodz University. This demonstrates that the University of Eastern Finland establishes its position as an absolute leader in this academic field. However, as the top two contributing countries, the institutions from the United States and China only rank the fourth (National Eye Institute, 22 papers) and the ninth (Sun Yat-Sen University, 15 papers), respectively. The cumulative number of publications over the years for the top five research institutions in the field of “AMD and autophagy” from January 2010 to October 2024 was shown as a line chart in Figure 4(b). The University of Eastern Finland had published 22 articles in this research field by the end of 2015, and since then, the number of publications has grown rapidly each year, reaching a total of 119 articles to date. The three-field plot shows the collaborative nature of research and examines the extent of collaboration among authors, institutions, and academic journals. As illustrated in Figure 4(c), the largest number of citations in the “Author (AU)” category is directed toward the University of Eastern Finland, indicating its significant position in the field of in the field of “AMD and autophagy” research.

### 3.4. Analysis of Journals

**Figure 5:**
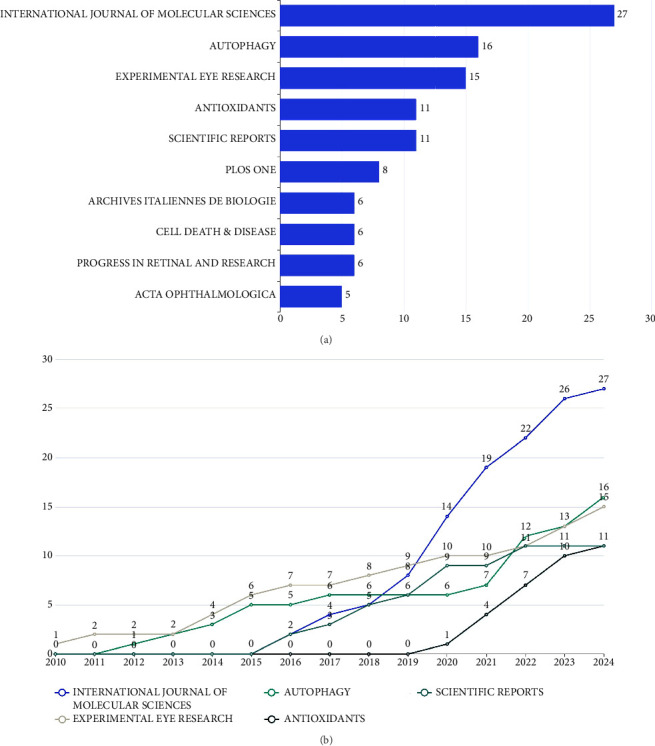
Analysis of journals on AMD and autophagy. (a) The leading 10 academic journals with the highest productivity in the research area of AMD and autophagy. (b) The total number of publications from 2010 to 2024 for the leading five academic journals focusing on AMD and autophagy.

A total of 141 academic journals published 349 publications. As shown in Figure 5(a), the top 10 journals contributed 111 publications, representing 31.81% of the total output. The International Journal of Molecular Sciences led in productivity with 27 publications, followed by Autophagy with 16 and Experimental Eye Research with 15. The cumulative number of publications over the years for the top five academic journals in the field of “AMD and autophagy” from January 2010 to October 2024 was shown as a line chart in Figure 5(b). This suggests that the aforementioned journals hold significant academic influence in the “AMD and autophagy” field.

We then investigated the top 10 journals according to the publication countries, impact factor (IF), and journal citation reports (JCRs), as illustrated in Table 1. Of the top 10 journals, 60% were originated from the United States and the United Kingdom. Based on JCR, 90% of the journals were classified as Q1, while 10% (specifically Archives Italiennes De Biologie, Italy) were classified as Q4.

### 3.5. Analysis of Authors

**Figure 6:**
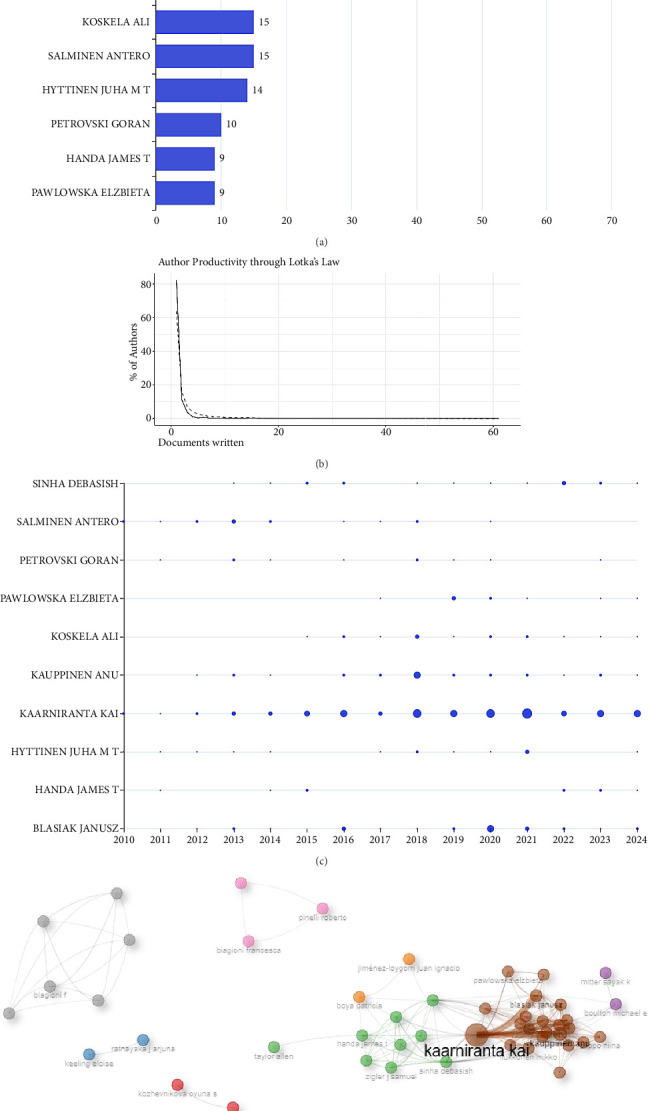
Analysis of authors on AMD and autophagy from 2010 to 2024. (a) The top 10 authors with the highest number of publications in AMD and autophagy research. (b) The author's productivity based on Lotka's law. (c) The top 10 most productive authors' output over time. The *x* axis and *y* axis represent the year and author, respectively. The larger the node, the more the publications. (d) The co-authorship network of 48 authors with more than two publications. A node's size represents an author's publication count. Clusters sharing the same color indicate the higher levels of collaboration.

The 349 publications come from 1767 researchers on AMD and autophagy from 2010 to 2024. As shown in Figure 6(a), the top 10 authors contributed 196 articles, representing 56.16% of the total publications. Kaarniranta Kai from the University of Eastern Finland leads with 61 papers, followed by Blasiak Janusz from the University of Lodz with 24 papers, and Kauppinen Anu, also from the University of Eastern Finland, with 23 papers, demonstrating their substantial impact on the field.

Furthermore, Lotka's law was applied to analyze the author's productivity. As shown in Figure 6(b), the frequency of single-article authors is the highest (82%), with a theoretical value of 64%, suggesting that the vast majority of authors published only one article on AMD and autophagy from 2010 to 2024. Simultaneously, a small number of authors published at least 10 or more publications (*n* = 8, 0.45%). This result aligns with the principles of Lotka's law, which states that most researchers are less prolific, contributing only a single publication to the field, yet a minority of authors produce the majority of scholarly literature studies.

A detailed overview of each author's publication timeline is shown in Figure 6(c). Darker-colored nodes represent a higher number of publications. Moreover, Figure 6(d) shows a co-authorship network comprising 48 authors, each with over two publications, highlighting significant collaborative efforts among various groups in AMD and autophagy research.

### 3.6. Analysis of References

The frequency of citations is indicative of the extent to which research findings on AMD and autophagy are acknowledged within their field. It serves as a crucial metric for assessing the study's quality.  Table 2 provides the detailed information of 10 publications with the most citations. The paper with the highest total number of citations (TC) was authored by Nita M and published in 2016 in Oxidative Medicine and Cellular Longevity, followed by Kaarniranta K's 2016 publication in Cellular and Molecular Sciences and Mitter SK's 2014 paper in Autophagy.

**Figure 7:**
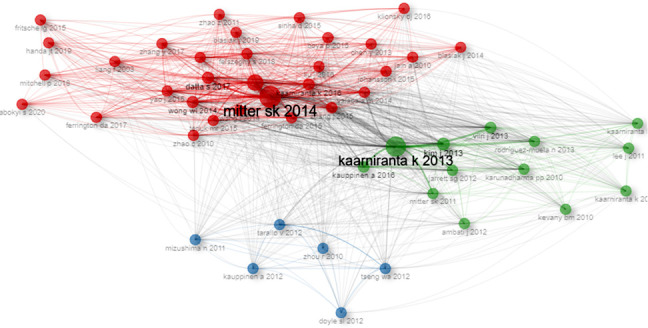
The cocitation network analysis map of the global literature in the field of AMD and autophagy study from 2010 to 2024.

The phenomenon of cocitation, wherein two scholarly works are collectively referenced within a single document, signifies a relationship between the publications. Such an analysis of cocitation references is instrumental in establishing the foundational knowledge structure of a scholarly domain. As shown in Figure 7, the cocitation network analysis of the global literature on AMD and autophagy from 2010 to 2024 reveals the core documents and their positions within the academic network. Mitter SK 2014 and Kaarniranta Kai 2013, as the most influential document, demonstrate their central role in the research literature. These findings not only uncover the key contributors to “AMD and autophagy” research but also reflect the interrelationships and distribution of influence among research topics.

### 3.7. Keyword Analysis

**Figure 8:**
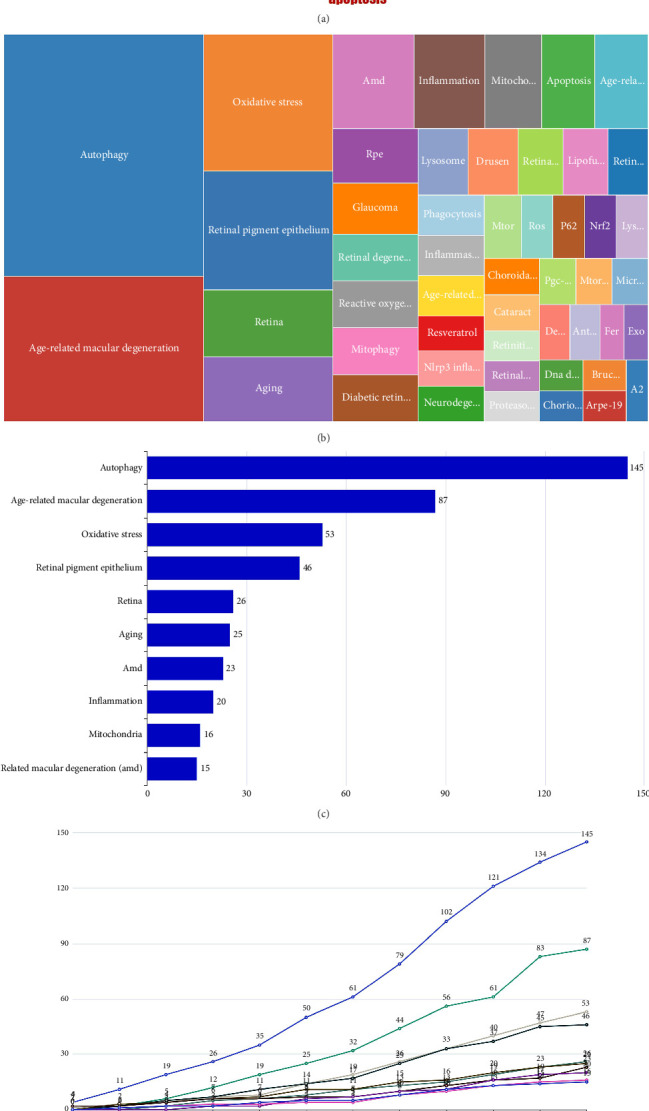
Analysis of keywords on AMD and autophagy from 2010 to 2024. (a) A word cloud map displays the top 50 keywords frequently used in the “AMD and autophagy” field, with font size representing their occurrence frequency. (b) The treemap displays the top 50 keywords most frequently used in “AMD and autophagy” research, with module areas representing their occurrence frequency. (c) The top 10 most frequently used keywords. (d) A line chart depicting the temporal frequency of the top 10 keywords in “AMD and autophagy” research.

A total of 751 keywords of the 349 articles were identified.  Figure 8(a) and 8(b) present a word cloud and a treemap, respectively, depicting the top 50 keywords frequently used in the “AMD and autophagy” research area. In the word cloud, font size reflects keyword frequency, while in the treemap, the module area represents the same. As illustrated in Figure 8(c), the top 10 most frequently used keywords were listed, with “autophagy” being the most frequent at 145 occurrences, followed by “age-related macular degeneration” (*n* = 87), “oxidative stress” (*n* = 53), “retinal pigment epithelium” (*n* = 46), “retina” (*n* = 26), “aging” (*n* = 25), “'amd” (*n* = 23), “inflammation” (*n* = 20), “mitochondria” (*n* = 16), and “age-related macular degeneration (amd)” (*n* = 15). These keywords highlight the key areas of focus in this research field.  Figure 8(d) presents a line chart illustrating the temporal frequency of the top 10 keywords in the relevant literature. “Autophagy” and “age-related macular degeneration” were the top two keywords in almost all periods, showing the dominance of these two keywords when taking the time dimension into the analysis. Recently, “oxidative stress” and “retinal pigment epithelium” have presented an upward trend.

## 4. Discussion

Autophagy is crucial for cellular maintenance by recycling damaged or unused components and is vital in the pathogenesis of various human diseases, such as cancer, diabetes, neurodegenerative disorders, and specific infections [20–22]. Recent studies suggest that impaired autophagy in RPE cells is crucial in AMD development and has gained significant attention as both a pathological mechanism and a potential therapeutic target [23].

This is the first in-depth bibliometric study of autophagy and AMD. A total of 349 publications spanning 2010 to 2024 were retrieved from the MEDLINE database. The annual publication counts gradually increased from 2010 to 2019, followed by a rapid growth period starting in 2020, reaching its peak in 2022 with over 40 publications. The growing volume of research papers highlights the sustained and increasing interest in the role of autophagy in AMD.

Publications retrieved from the MEDLINE database are contributed from 33 countries. Research on autophagy and AMD in China began since 2014, which was later than other leading countries, yet it subsequently experienced rapid growth, quickly becoming a major contributor to the field. Despite a high level of interest in this field among Chinese scholars, only one Chinese institution appears in the top 10 for publication volume with 15 articles (Sun Yat-Sen University). Additionally, the average citation count is relatively low. Still, the academic influence of these studies is limited. Therefore, while China has demonstrated rapid growth in publication numbers, the relatively lower citation impact suggests a need for enhancing research visibility and quality. Strengthening international collaborations, fostering cross-country exchange of expertise, and aligning with established research centers worldwide may help to increase the global influence of this field in a more evidence-based and sustainable manner. The United States leads in the TC and pioneered research in this field, although ranking the second in the number of published articles among the top 10 most prolific countries [24]. An analysis of institutions reveals that the United States holds the highest representation in the top 10 for the number of published papers, occupying four positions. This demonstrates the United States's significant academic influence and robust support for scholars in autophagy research related to AMD, highlighting the collaborative efforts among scholars from various institutions. Finland ranks third in article contributions behind China and the United States, yet surpasses China in the TC. The University of Eastern Finland leads in both institutional publications and individual author contributions. Overall, Finland provided an important platform for subsequent research by various scholars in the field of autophagy in AMD [25].

Ninety percent of the leading 10 high-yield journals are classified as JCR Q1, indicating their significant academic reputation in the field. The International Journal of Molecular Sciences, which started publishing the related literature in 2016, has rapidly increased the number of publications in recent years, becoming the leading journal in terms of article volume and reflecting its recognition and trust among authors. Although the number of articles published in Autophagy ranked second, there have been continuous articles published since 2011, and the IF is significantly higher than that in the International Journal of Molecular Sciences, indicating that the articles published in Autophagy have a higher academic level. Focusing on these journals grants access to the most advanced and impactful research in the field.

References form the foundation of research, and analyzing them through cocitation or citation methods aids in identifying the knowledge repository of a specific research area. The paper by Małgorzata Nita, published in Oxidative Medicine and Cellular Longevity in 2016, has the highest citation count [26]. It demonstrates that oxidative stress levels rise in the aging retina, leading to chronic inflammation. Inflammation, coupled with heightened metabolic activity, results in increased oxygen consumption and subsequent retinal hypoxia. Chronic oxidative stress, pathophysiological parainflammation, and prolonged hypoxia diminish the autophagy capacity of RPE cells, significantly contributing to the onset and progression of AMD. The second most cited article was from Kaarniranta Kai, who is the most prolific author, contributing 61 articles [27]. The article confirmed chronic inflammation's role in AMD pathogenesis and detailed the inflammatory processes and mechanisms at each stage of the disease. Although the publication authored by Mitter in 2014 [13], which appeared in Autophagy, held the third ranking in terms of citations, it was the most cocited paper. Mitter et al. highlight the crucial role of autophagy in safeguarding RPE from oxidative stress and lipofuscin buildup, noting that its dysfunction may intensify oxidative stress and aid in AMD pathogenesis. Together, these studies indicate that autophagy, in conjunction with inflammation and oxidative stress, contributes to AMD development.

Keywords in the academic literature typically indicate research field trends. The research encompasses 751 keywords, with the top 10 high-frequency terms being autophagy, AMD, oxidative stress, RPE, retina, aging, AMD, inflammation, and mitochondria. From the results of key stems, we can see that the crosstalk between autophagy and oxidative stress as well as inflammation may be a novel field. RPE cell impairment is the core event in AMD and exhibits a strong susceptibility to oxidative stress due to their abundant oxygen levels, vigorous metabolism, and direct contact with light sources [28, 29]. Oxidative stress influences various stages of autophagy [29–31], and the age-related decline in the phagocytic function of RPE cells further diminishes autophagy levels [32]. Regulating autophagy to reduce oxidative damage and targeting the removal or suppression of senescent RPE cells have emerged as potential therapeutic strategies for AMD in recent years. In addition, excessive reactive oxidative stress and the accumulation of cellular waste can cause the production of local inflammatory factors and finally lead to RPE cell damage and even death [33, 34]. As a protective mechanism, autophagy has not been found to play a certain regulatory role in the immune inflammation response in AMD. Therefore, subsequent research should focus on specific inflammatory biomarkers and the regulatory role of autophagy on inflammation. Due to its high energy requirements, the retina is prone to mitochondrial imbalances, in which mitochondrial dysfunction and mitochondrial DNA/mtDNA damage are observed in AMD [12]. Mitophagy selectively removes damaged mitochondria via autophagy, essential for mitochondrial homeostasis [35, 36]. Till now, there are very few reports on mitophagy. Thus, focusing on mitophagy could offer a novel strategy for AMD treatment.

This study utilized bibliometrics to visualize research on autophagy and AMD, enhancing the understanding of key areas and trends in the field. However, it suffers from many limitations. Firstly, owing to software limitations, only articles in the MEDLINE database are encompassed in this paper. This will decrease the number of articles and citations, which might impact the final analysis result. Future analyses should incorporate additional databases for increased rigor. Secondly, only articles and reviews published in English are incorporated in this paper, which will lead to the omission of a considerable amount of non-English literature and result in a certain publication bias. Finally, the continuous publication of the literature will result in the continuous update of data and lead to different analysis conclusions. Consequently, the current bibliometric analysis research design requires enhancement due to the limitations of the existing methods.

Through this analysis, we know that the related research on autophagy in AMD is still in an upward trend. China leads in productivity in this research area, whereas the United States holds the most influence. Although Finland ranks third after China and the United States in the number of publications. The institutions and authors with the most publications are also from Finland. The most frequently cited studies primarily examine the interplay between inflammation, oxidative stress, and autophagy in RPE cells, suggesting that future research will likely concentrate on how autophagy regulates inflammation and oxidative stress. Keyword analysis revealed growing interest in mitophagy alongside inflammation and oxidative stress, suggesting that further research and regulation of mitophagy could offer a novel approach for AMD treatment.

## 5. Conclusions

This bibliometric analysis identifies current research hotspots in autophagy related to AMD and informs future research directions. Future trends in this field may involve identifying and developing novel autophagy-targeted therapies for the prevention and treatment of AMD.

## Figures and Tables

**Table 1:** Top 10 journals with the most publications in the field of AMD and autophagy.

Rank	Journal	Articles	Country	If (2023)	JCR-c
1	International Journal of Molecular Sciences	27	Switzerland	4.9	Q1
2	Autophagy	16	USA	14.6	Q1
3	Experimental Eye Research	15	USA	3	Q1
4	Antioxidants	11	Switzerland	6	Q1
5	Scientific Reports	11	UK	3.8	Q1
6	PLoS One	8	USA	2.9	Q1
7	Archives Italiennes De Biologie	6	Italy	0.8	Q4
8	Cell Death & Disease	6	UK	8.1	Q1
9	Progress In Retinal And Eye Research	6	UK	18.6	Q1
10	Acta Ophthalmologica	5	Denmark	3	Q1

Abbreviations: IF, impact factor; JCR, journal citation report; USA, United States of America; UK, United Kingdom.

**Table 2:** Top 10 cited references regarding the research on AMD and autophagy from 2010 to 2024.

Rank	PMID number	Year	Journal	If (2023)	Title	Author	TC
1	26881021	2016	Oxidative Medicine and Cellular Longevity	N/A	The Role of the reactive oxygen species and oxidative stress in the pathomechanism of the age-related ocular diseases and other pathologies of the anterior and posterior eye segments in adults.	Nita M	466
2	26852158	2016	Cellular and Molecular Sciences	6.2	Inflammation and its role in age-related macular degeneration.	Kaarniranta K	303
3	25484094	2014	Autophagy	14.6	Dysregulated autophagy in the RPE is associated with increased susceptibility to oxidative stress and AMD.	Mitter SK	249
4	29988039	2018	Cell Death & Disease	8.1	Glutathione depletion induces ferroptosis, autophagy, and premature cell senescence in retinal pigment epithelial cells.	Sun Y	197
5	28055007	2017	Cell Death & Disease	8.1	Dysfunctional autophagy in RPE, a contributing factor in age-related macular degeneration.	Golestaneh N	180
6	21559389	2011	PLoS One	2.9	Age-related retinopathy in NRF2-deficient mice.	Zhao Z	178
7	23590900	2013	Autophagy	14.6	Autophagy and heterophagy dysregulation leads to retinal pigment epithelium dysfunction and development of age-related macular degeneration.	Kaarniranta K	173
8	23220125	2013	Biochimica et Biophysica Acta (BBA)—Molecular Cell Research	4.6	Maturation of autophagosomes and endosomes: a key role for Rab7.	Hyttinen JM	170
9	24335072	2013	Investigative Ophthalmology & Visual Science	5	Dry age-related macular degeneration: Mechanisms, therapeutic targets, and imaging.	Bowes Rickman C	129
10	26344735	2015	Progress In Retinal and Eye Research	18.6	Defects in retinal pigment epithelial cell proteolysis and the pathology associated with age-related macular degeneration.	Ferrington DA	118

Abbreviations: IF, impact factor; TC, total number of citations; N/A, not available.

## Data Availability

All data generated or analyzed during this study are included in this published article, and further inquiries can be directed to the corresponding author.

## Nomenclature

AMD: age-related macular degeneration
ARPE-19 cell: Adult Retinal Pigment Epithelial Cell Line-19 cell
CNV: choroidal neovascularization
GA: geographic atrophy
IF: impact factor
JCR: journal citation report
RPE: retinal pigment epithelium
TC: total number of citations
UK: United Kingdom
USA: United States of America
VEGF: vascular endothelial growth factor
